# Co-occurrence of Distinct Ciliopathy Diseases in Single Families Suggests Genetic Modifiers

**DOI:** 10.1002/ajmg.a.34173

**Published:** 2011-10-14

**Authors:** Maha S Zaki, Shifteh Sattar, Rustin A Massoudi, Joseph G Gleeson

**Affiliations:** 1 Clinical Genetics Department, Human Genetics and Genome Research Division, National Research CentreCairo, Egypt; 2 Neurogenetics Laboratory, Department of Neurosciences and Pediatrics, Howard Hughes Medical Institute, University of CaliforniaSan Diego, California

**Keywords:** molar-tooth, polydactyly, intellectual disability, retinal blindness, obesity, nephronophthisis

## Abstract

Disorders within the “ciliopathy” spectrum include Joubert (JS), Bardet–Biedl syndromes (BBS), and nephronophthisis (NPHP). Although mutations in single ciliopathy genes can lead to these different syndromes between families, there have been no reports of phenotypic discordance within a single family. We report on two consanguineous families with discordant ciliopathies in sibling. In Ciliopathy-672, the older child displayed dialysis-dependent NPHP whereas the younger displayed the pathognomonic molar tooth MRI sign (MTS) of JS. A second branch displayed two additional children with NPHP. In Ciliopathy-1491, the oldest child displayed classical features of BBS whereas the two younger children displayed the MTS. Importantly, the children with BBS and NPHP lacked MTS, whereas children with JS lacked obesity or NPHP, and the child with BBS lacked MTS and NPHP. Features common to all three disorders included intellectual disability, postaxial polydactyly, and visual reduction. The variable phenotypic expressivity in this family suggests that genetic modifiers may determine specific clinical features within the ciliopathy spectrum. © 2011 Wiley Periodicals, Inc.

## INTRODUCTION

Ciliopathies are recessively inherited conditions that share at their core some dysregulation of structure or function of the cellular primary cilium. Ciliopathies commonly include Leber congenital amaurosis (LCA, i.e. congenital retinal blindness (OMIM #204000), nephronophthisis (NPHP, OMIM #256100), and Joubert syndrome (JS, OMIM #213300). LCA is characterized by congenital blindness due to defective photoreceptors, NPHP is characterized by a fibrocystic transformation of the kidney, whereas JS is characterized by the presence of a pathognomonic “molar tooth” sign (MTS) of the midbrain–hindbrain junction on axial brain imaging. Several overlapping syndromes have been described including Senior–Loken syndrome (LCA + NPHP, OMIM #266900), and cerebellooculorenal syndrome (JS + LCA + NPHP, OMIM #608091) [Valente et al., 2003], suggesting either unique syndromes or variable expressivity.

Bardet–Biedl syndrome (BBS, OMIM #209900) is included within the ciliopathy spectrum of disorders, characterized by major criteria of rod–cone retinal dystrophy, postaxial polydactyly, truncal obesity, learning disabilities, hypogonadism, and renal anomalies. Several disorders overlapping with BBS have emerged including MORM (mental retardation, truncal obesity, retinal dystrophy, and micropenis, OMIM #610156), Alström (rod–cone dystrophy, sensorineural hearing loss, obesity, hyperinsulinemia, and type 2 diabetes, OMIM #203800), and Biedmond 2 (iris coloboma, intellectual disability, obesity, hypogenitalism, postaxial polydactyly OMIM #210350) syndromes.

Despite this syndromology, there is extensive clinical overlap between these entities. For instance, all of the ciliopathies can be accompanied by intellectual disability, retinopathy, polydactyly, and hepatic diseases, making it a challenge to define the limits of each syndrome [Gerdes et al., 2009]. In addition to this clinical heterogeneity is the finding of molecular heterogeneity. Each ciliopathy syndrome can be caused by mutations in any one of a number of genes, and a given gene implicated in the ciliopathies has frequently been associated with several different syndromes. The best example is for *CEP290*, mutations of which have been identified in patients with LCA, NPHP, SLS, JS, BBS, and Meckel–Gruber syndrome (MKS) [Coppieters et al., 2010]. *TMEM67* mutations can cause BBS, JS, and MKS, with a particular predilection for liver involvement [Smith et al., 2006; Leitch et al., 2008; Brancati et al., 2009]. *INPP5E* mutations are associated with both MORM and JS [Bielas et al., 2009; Jacoby et al., 2009]. Importantly, even though the phenotypic spectrum associated with most ciliopathy genes is widely divergent, the spectrum reported within a given multiplex family has largely been limited to variable polydactyly or retinopathy [Valente et al., 2006a; Brancati et al., 2007; Otto et al., 2008].

Here we report two consanguineous Egyptian families each with non-overlapping ciliopathy spectrum disorders. In Family Ciliopathy-672, one child displayed NPHP and polydactyly but not the MTS, whereas another child displayed only the MTS but not NPHP. In a second branch of this family, two other children displayed NPHP but not the MTS. In Family Ciliopathy-1491, one child displayed classical features of BBS but not the MTS, and the two other children displayed the MTS without classic features of BBS. Variable degrees of cognitive impairment presented in all three children, and polydactyly was present in two, suggesting a common genetic etiology. These families highlight the degree of variable expressivity that can be observed within a single family within the ciliopathy spectrum, and suggest genetic modifiers might be responsible for these differences.

## CLINICAL REPORT

### Family 1

**FIG. 1:**
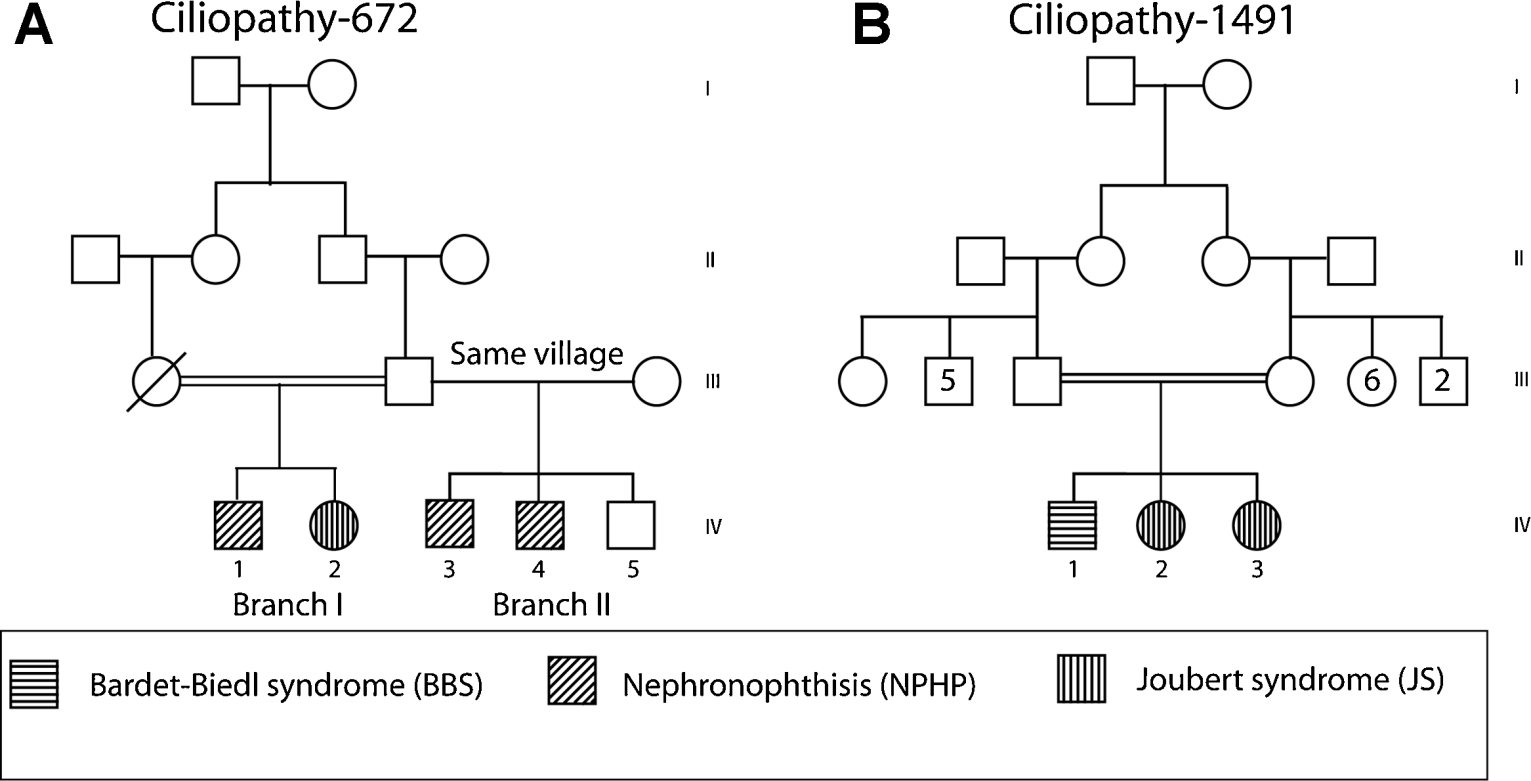
A: Pedigree of ciliopathy-672, showing first cousin marriage with two affected members in Branch I, NPHP in IV-1, and JS in IV-2. After the death of the mother of Branch I, the father remarried (Branch II) and had three boys, two affected with NPHP. **B**: Pedigree of ciliopathy-1491, showing first-cousin marriage and three affected children. The oldest displays classic features of BBS, and the younger two display the MTS.

**FIG. 2:**
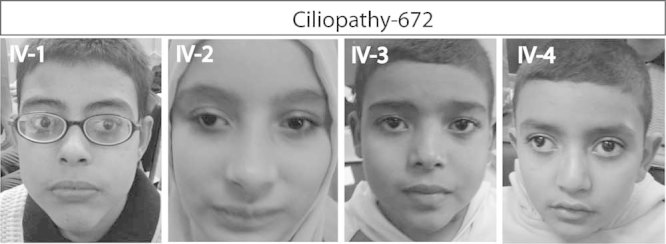
Facial appearance of affected members from Ciliopathy-672, showing absence of dysmorphic features.

The family Ciliopathy-672 is from Upper Egypt and has two branches (Figs. 1, 2, and 4) (Table I). In Branch 1, a first cousin marriage produced two children. The proposita was a 14-year-old female (IV-2) who presented with mild intellectual disability and wide base gait. On examination, she had autistic manifestations, oculomotor apraxia, nystagmus, squint, and hypotonia. Investigations including fundus examination, VEP, ERG, kidney, and liver function tests and abdominal sonar were normal, whereas brain MRI showed MTS. Family history and pedigree analysis verified a male brother 18 years old (IV-1) with below average intelligence and renal failure. The renal symptoms started at 10 years with polyuria/polydypsia and elevated serum creatinine. The condition was compensated until age of 13 when dialysis was required. Abdominal sonar showed small atrophic kidneys, increased echogenicity, and prominent corticomedullary cysts. Histological findings of the kidney biopsy were consistent with nephronophthisis. Physical examination revealed mild hypotonia, unilateral postaxial polydactyly and nystagmus with visual reduction although fundus examination and ERG were normal. MRI showed mild cerebellar vermis hypoplasia without the MTS evident (Supplemental Fig. 1). After the death of the mother, the father remarried a woman from the same small village, suggesting she is a carrier of the same mutation. The first two boys (aged 12 and 10 years, respectively) developed NPHP around the age of 10 years, but neither is dialysis-dependent currently. However, serum creatinine was 6.8 mg/dl for the older child (reference 0.5–1.0 mg/dl) and he was scheduled for dialysis while the younger had serum creatinine 1.6 mg/dl and still compensated. Examination for these two siblings was non-contributory except for growth failure in addition to visual reduction in IV-3 and mild intellectual disability in IV-4. Renal ultrasounds showed normal sized kidneys but increase echogenicity and poor corticomedullary differentiation with occasional renal cysts. Fundus examination and ERG were normal for both of them and MRI showed normal brain structure in IV-3 and IV-4.

**TABLE I:** Clinical Findings of the Present 2 Families

	Family Ciliopathy-672				Family Ciliopathy-1491		
		
	IV-1	IV-2	IV-3	V-4	IV-1	IV-2	IV-3
Age	18y	14y	12y	10y	6y	5y	3y
Sex	M	F	M	M	M	F	F
Height	148 cm (−3.9SD)	146 cm (−2.3SD)	120.5 cm (−4SD)	110 cm (−4.1SD)	123 cm (+1.7 SD)	104 cm (−1.2SD)	80.5 cm (−3.2SD)
Weight	38 kg (−2.6SD)	35 kg (−1.8SD)	22 kg (−1.6SD)	18 kg (−1.7SD)	46 kg (+4.7SD)	18 kg (−0.4SD)	10 kg (−2.6SD)
Head circumference	54 cm (−USD)	53 cm (mean)	52 cm (−1SD)	51.5 cm (0.5SD)	54 cm (+1.6SD)	50 cm (−0.3SD)	46.5 (−2.3SD)
Karyotype	46 XY	46 XX	46 XY	46 XY	46 XY	46 XX	46 XX
Neurological signs							
Hypotonia	Y	Y	N	N	Y	Y	Y
Mental retardation	Moderate IQ72	Moderate 16	N IQ95	Moderate IQ82	Mild IQ74	Moderate 16	Moderate IQ40
OMA	Y	Y	N	N	N	Y	Y
Breathing abnormalities	M	N	N	N	N	Y	Y
Ocular signs							
Visual reduction	Y	Y(mild)	N	Y	Y	N	N
Coloboma	N	N	N	N	N	N	N
Corneal opacity	N	N	N	N	N	N	Y
Squint	mild	Y	N	Y	N	Y	Y
Nystagmus	Y	Y	N	Y	Y	N	N
Pigmentary retinal changes	M	N	N	N	Y	N	N
Abnormal Electroretinogram	N	N	N	N	Y	M	N
Renal signs							
NPHP	Y (biopsy+)	N	Y	Y	N	M	N
Polydypsia	M/A	N	Y	Y	Y	M	N
Creatinine (mg/dl)	N/A	N	6.8	1.6	N/A	N/A	N/A
Dialysis	Y	N	N	N	N	N	N
Other signs							
Obesity	N	N	N	N	Y	N	N
Postaxial polydactyly	UUE	N	N	N	BUE/BLE	Y	RUE
Liver fibrosis	N	N	N	N	N	N	N
Self-mutilation	N	N	N	N	N	Y	Y
Penile length	10 cm	N/A	4.5 cm	4 cm	2.5 cm	N/A	N/A
Brain MRI reading							
Molar tooth sign	M	Y	N	N	N	Y	Y
Cerebellar vermis hypoplasia	Mild	Y	N	N	Mild	Y	Y

Abnormal electroretinogram indicates evidence of rod–cone dystrophy; BUE, bilateral upper extremity; BLE, bilateral lower extremity; IQ, intelligent quotient; N/A, not available or not applicable; OMA, oculomotor apraxia; NPHP, nephronophthisis; RUE, right upper extremity.

### Family 2

**FIG. 3:**
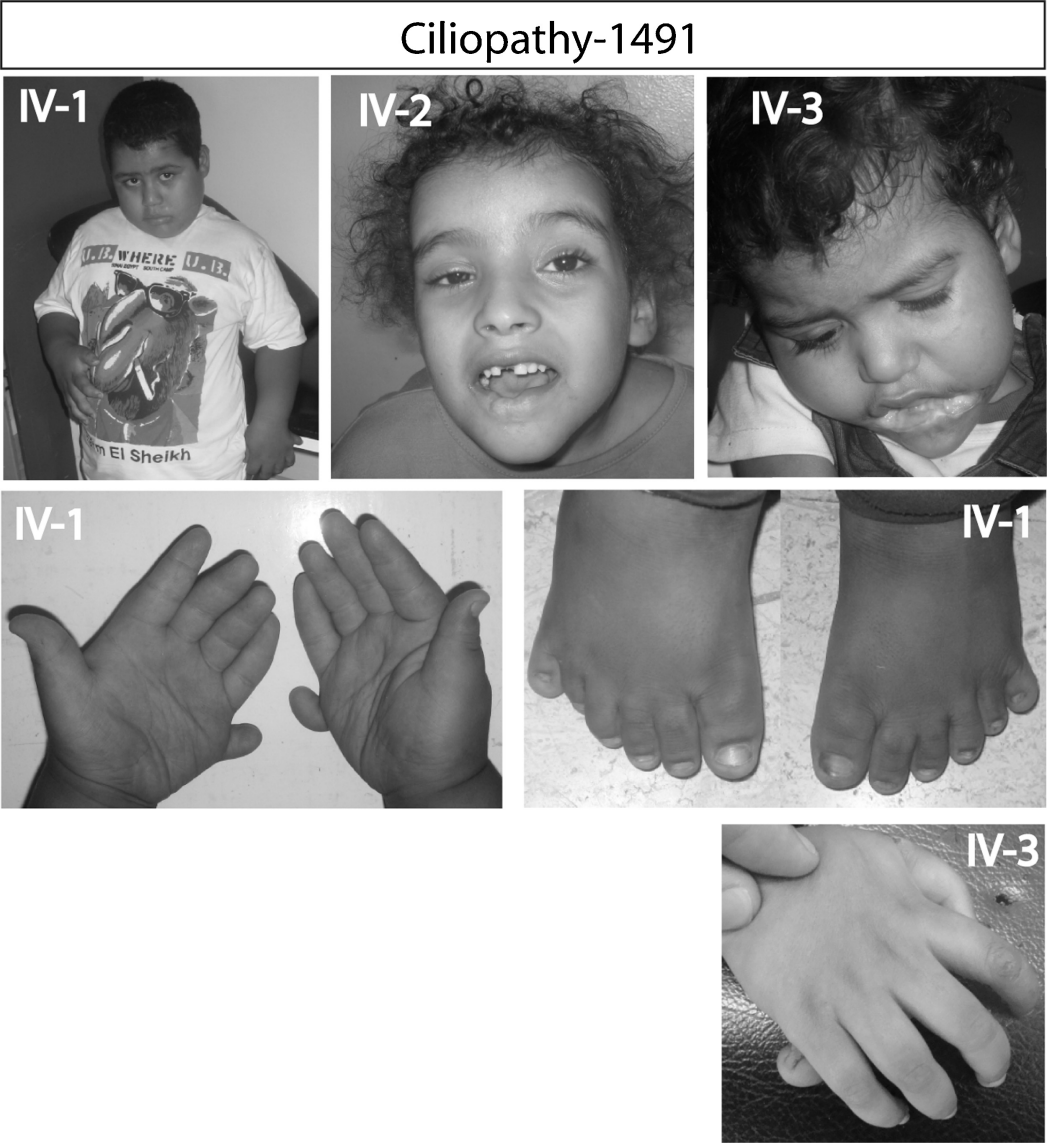
Clinical features from Ciliopathy-1491. IV-1 shows obesity and bilateral upper and lower extremity postaxial polydactyly. IV-2 shows ptosis of the right eye. IV-3 shows self-inflicted abrasions of the perioral region and postaxial polydactyly of the right hand only.

**FIG. 4:**
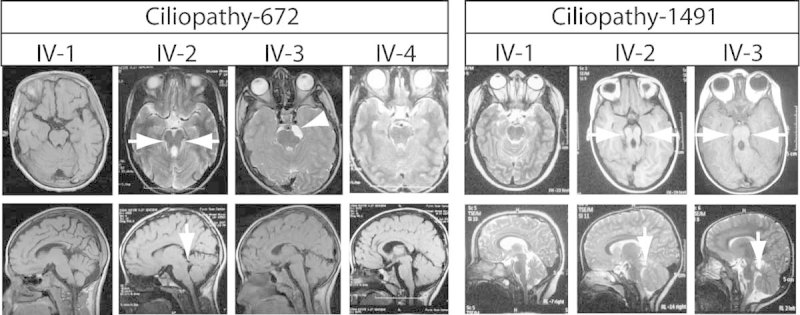
Brain MRI of affected members of both families (axial view at the midbrain-hindbrain junction (top) and midline sagittal (bottom)). In Ciliopathy-672, MTS is evident only in IV-2 (arrows), and is absent in IV-1, -3, and -4. There is a small arachnoid cyst evident in IV-3 (arrowhead), not seen in the other members. Consistent with the axial views, only IV-2 shows a horizontally oriented superior cerebellar peduncle (arrow). Despite the absence of the MTS, there is mild cerebellar affection at lest for IV-1. In Ciliopathy-1491, MTS is evident in IV-2 and -3 (arrows) evidence by the horizontally oriented superior cerebellar peduncle (arrows). In IV-1 there is mild cerebellar vermis hypoplasia without the MTS.

The Family Ciliopathy-1491 is from Upper Egypt, with one affected boy and two affected girls (Figs. 1, 3, and 4) (Table I). The parents are healthy first cousins. There are no healthy children in the family. The family history is otherwise negative, with a total of 14 aunts or uncles, arguing against dominant inheritance. The family presented to Neurogenetics Clinic due to familial intellectual disability. All had delayed physical and developmental milestones. The oldest child (IV-1) was a 6-year-old male with classic features of BBS. He had intellectual disability, obesity (height 123 cm (+1.7 SD), weight 46 kg (+4.7 SD), body mass index 31.9 SI units) and was unable to see objects at night. On examination, the patient had squint strabismus with nystagmus, hypotonia and bilateral postaxial polydactyly in both upper and lower limbs. Investigations were normal concerning liver and kidney function tests and abdominal ultrasound. Brain MRI showed mild cerebellar vermis hypoplasia without the MTS evident. Pigmentary retinal changes were detected on fundus examination. Electroretinogram showed evidence of rod–cone dystrophy.

The second child (IV-2) was a 5 4/12-year-old female with anthropometric measurements of −1.2 SD for height, −0.4 SD for weight, and −0.3 SD for OFC. She was able to walk with wide base gait and acquired linguistic skills with few words. Patient had mild autistic behavior, oculomotor apraxia, squint strabismus, nystagmus, and hypotonia with history of abnormal breathing patterns and some attacks of self-mutilation such as biting tongue and lips. No obesity or polydactyly were observed. Brain MRI showed the MTS, constituted by cerebellar vermis hypoplasia/dysplasia and thickened elongated and horizontally oriented superior cerebellar peduncles. Other investigations were noncontributory including fundus examination, ERG, liver and kidney function tests, and abdominal sonar.

The youngest girl (IV-3) was 3 2/12-years-old with history of recurrent attacks of extreme self-mutilation. On examination, patient could sit and vocalize with few single syllable words. She had failure to thrive. Her height was 80.5 cm (−3.2 SD), weight was 10 kg (−2.6 SD), and OFC was 46.5 cm (−2.3 SD). Autistic behavior, excessive biting of lips and hands, and abnormal breathing patterns were noted. Patient had oculomotor apraxia, nystagmus, small bilateral corneal opacity (developed one year ago), right postaxial polydactyly, and hypotonia with preserved reflexes. Because of corneal opacity and self-mutilation, metabolic screening testing and homovanillic acid/vanillylmandelic acid ratio were performed to exclude tyrosinemia II and familial dysautonomia, respectively. Brain MRI showed clear MTS whereas kidney and liver function tests, abdominal sonar, and fundus examination, ERG was normal.

## METHODS

Patients were ascertained according to approved protocols at National Research Centre, Egypt and the University of California, San Diego. Signed consent was obtained from all participants, and consent was further obtained to allow for reproduction of facial photographs in scientific manuscripts. Intelligence testing was performed using the Stanford–Binet Scale. DNA was extracted from peripheral blood lymphocytes using Qiagen Maxiprep DNA extraction method. PCR was performed using reagents from Qiagen, and DNA sequencing was performed on an ABI 3700 Capillary Sequencer.

## RESULTS

There have been six genes reported to cause both NPHP and JS: *NPHP1*, *AHI1*, *CEP290*, *RPGRIP1L*, *TMEM67*, and *TMEM216* [Hildebrandt et al., 1997; Dixon-Salazar et al., 2004; Ferland et al., 2004; Parisi et al., 2004; Sayer et al., 2006; Utsch et al., 2006; Valente et al., 2006b; Arts et al., 2007; Baala et al., 2007; Delous et al., 2007; Otto et al., 2009; Valente et al., 2010]. In each case, the gene is primarily associated with one of the two disorders but some percentage of patients with mutations display features of both diseases. In order to exclude Ciliopathy-672 from linkage to any of these loci, we performed SNP genotyping using markers surrounding each of the genes [Murray et al., 2004]. At least two polymorphic markers per gene (rs1718031 and rs729386 for *NPHP1*; rs1041480 and rs2064430 for *AHI1*; rs2061589 and rs1508595 for *CEP290*; rs1990637 and rs1946155 for *RPGRIP1L*; rs1483457 and rs911 for *TMEM67*) were inconsistent with linkage in Ciliopathy-672.

There have been three different genes reported to cause both BBS and JS: *CEP290*, *TMEM67*, and *INPP5E* (in the case of *INPP5E*, mutations are associated with the related disorder MORM syndrome, which is probably a phenocopy of BBS) [Baala et al., 2007; Leitch et al., 2008; Bielas et al., 2009; Jacoby et al., 2009]. Therefore, we considered it most important to exclude these three genes as causative in the Ciliopathy-1491 family, with discordant BBS and JS. We utilized a similar mapping strategy to exclude *CEP290* and *TMEM67* genes based upon heterozygosity in the affected members (marker Cr12-86.9 for *CEP290*, and D8S1794 for *TMEM67*). *INPP5E* could not be excluded using this strategy, but direct sequence analysis of all exons and splice sites excluded potentially deleterious sequence changes. Thus, we were not able to detect the genetic basis of the ciliopathy phenotype in these two families.

## DISCUSSION

We report on two families with at least partial discordance for ciliopathy phenotypes [Badano et al., 2006]. The ciliopathies are an expanding group of partially overlapping clinical entities, unified by the observation both of allelism between what were previously considered separate syndromic conditions, as well as finding that the protein products are localized predominantly to the basal body or cilium of cells from the affected organs [Ansley et al., 2003; Mykytyn and Sheffield, 2004].

Many previous studies have extended the phenotypic spectrum of given ciliopathy disorders; for example the description of retinopathy or NPHP in patients with JS, or MTS in some patients with NPHP, which have blurred the boundaries of what were initially described as unique syndromes, and led to a reclassification of how we consider the individual ciliopathy diseases [Cardenas-Rodriguez and Badano, 2009].

Concurrent with this blurring have been the many studies demonstrating allelism between these various conditions. One point of clarity came from the finding that mutation severity can predict disease severity for a given gene. For instance, null mutations in certain genes cause MKS, a lethal ciliopathy, whereas hypomorphic mutations give rise to the milder BBS [Leitch et al., 2008]. Similarly, null mutations in *CEP290* give rise to CORS whereas a specific intronic hypomorphic mutation is the most common cause of isolated LCA in Caucasians [den Hollander et al., 2006].

Keeping in mind that the ciliopathies are classically considered recessive conditions, and that both pedigrees display first-degree cousin consanguinity, the discordance for ciliopathy phenotypes within single families suggests one of several possible explanations, which are considered one-by-one:

- Single gene cause plus modifiers in each family.
- Multiple distinct genetic disorders occurring by chance in the same family.
- Inadequate phenotypic assessment.
- Oligogenic inheritance.

(a) The most likely possibility, it appears, would be that all patients within a single family have inherited the same mutation from each parent. This would result in a homozygous mutation in a ciliopathy gene, transmit as a simple recessive disease in a 1:3 ratio, and thus set the affectation status. What then would determine the variable expressivity? Presumably additional modifier genes that are present in the genome of one or both parents have some effect on the presence/absence of the unique features that determine the expressivity that is unique to the particular syndrome as we know it. Indeed, several modifiers have been identified to alter expressivity, notably to enhance the retinal or cerebral involvement in the ciliopathies. In these examples, a primary homozygous or compound heterozygous mutation sets affectation that is modified by the presence of polymorphism(s) in different gene(s). These examples include a common polymorphism in the *RPGRIP1L* gene that is associated with retinal disease across the ciliopathies [Khanna et al., 2009], a common polymorphism in *AHI1* that is associated with retinal disease in patients with nephronophthisis [Louie et al., 2010], and a worsened neurological phenotype in patients with a *CEP290* mutation together with a heterozygous *AHI1* mutation [Coppieters et al., 2010]. Although more work is needed to confirm and expand these studies, there is accumulating evidence that the ciliopathy genes work in concert to determine expressivity.

(b) Least likely seems the possibility that the discordance is due to two different primary mutations, such that each parent is heterozygous both for an NPHP and JS gene in the case of the first family, and both for a BBS and JS gene in the case of the second family. In this scenario, a family has two separate recessive diseases, each with a 25% recurrence risk. Given the rarity of this condition, the expected carrier frequencies would make this possibility unlikely, especially since the diseases have the same ciliary pathogenesis, but it could only be fully excluded by identifying the causative mutations.

(c) While we utilized careful clinical assessments and state-of-the-art diagnostic studies to evaluate each patient, it is important to point out that these tests have arbitrary thresholds for what is considered within the normal range. For instance, there is a range of what is considered normal in cerebellar size and kidney echogenicity on imaging studies, so it is possible that what is considered normal is in actuality subtly affected below current threshold. Further there is an age-dependence for onset of some of these features, for instance the retinal and renal features may not develop until the second decade. Finally, a normal fundus exam does not exclude subtle retinal dystrophy. For these reasons, it is impossible to be certain that the discordance is as absolute in these families as the clinical studies would suggest. In the future, it might be useful to perform quantitative assessments of these studies to evaluate for subtle differences once causative genes are identified.

(d) Although the ciliopathies are recessive conditions, some exceptions have been proposed in the form of oligogenic inheritance and double heterozygosity for BBS and retinitis pigmentosa (a late onset form of LCA), respectively [Kajiwara et al., 1994; Katsanis et al., 2001]. Thus, an alternative model must include the possibility of such complex genetic interactions. In this scenario, each parent would carry three or more unique mutations, and expressivity would be set by the cumulative sorting of the variants in terms of mutational load. This model is not as attractive as the simple modifier model, due to parent consanguinity in both pedigrees, but it has been verified experimentally in model systems [Williams et al., 2010].
